# Supplementary material for: Analyzing postprandial metabolomics data using multiway models: a simulation study
Source: BMC Bioinformatics. 2024 Mar 4;25:94. doi: 10.1186/s12859-024-05686-w (PMC10913623; doi:10.1186/s12859-024-05686-w)
Supplement: Supplementary file 2 — Additional file 2. Supplementary figures and tables for the main manuscript. [file 12859_2024_5686_MOESM2_ESM.pdf]

Supplementary figures and tables for the main manuscript

Supplementary figures

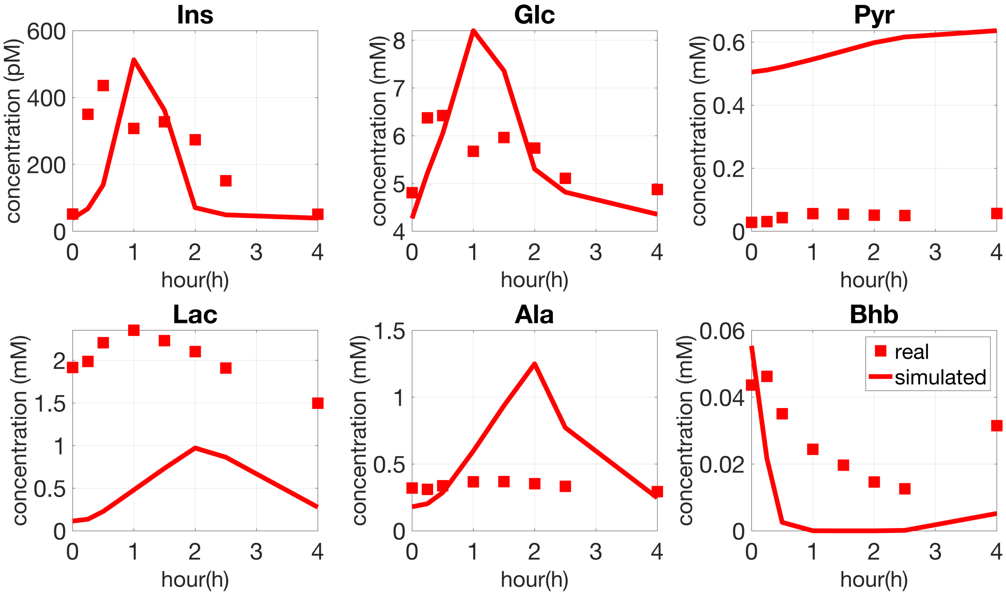

Figure S2.1: **Median time profiles of the real vs. simulated (50 control subjects and with the random perturbation level for individual variation set to $\alpha = 0.2$) data generated from the default human whole-body metabolic model.**

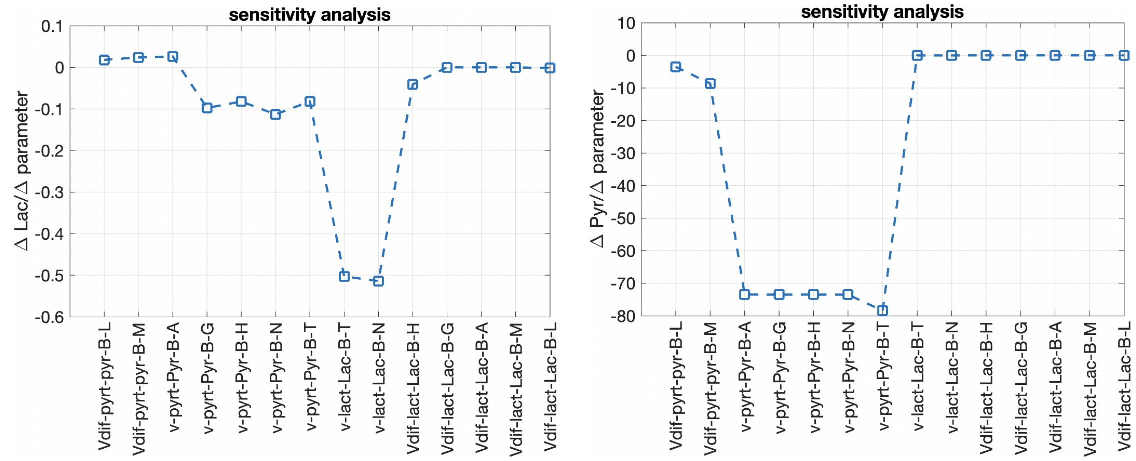

Figure S2.2: **Sensitivity analysis for metabolites Lac (left panel) and Pyr (right panel). We observed an apparent deviation of Pyr from the reference range $[0.04, 0.1]$ mmol/L using the default model, as shown in the figure in Additional file 1; therefore, we performed a sensitivity analysis (checking how the change of parameters related to Pyr will affect the change of its 10h-fasting concentration) to tune the parameters in the human whole-body model so that the 10h-fasting concentration of Pyr falls into the reference range. From Figure S2.2 (right panel), we select to increase v-pyrt-pyr-B-A by 0.007 (the default value is 0) to obtain a significant decrease of the 10h-fasting Pyr. In addition, we increase Vdif-pyrt-pyr-B-L by 0.003 (the default value is 0) to make an adjustment. Although the left panel in S2.2 indicates decreasing v-lact-lac-B-N or v-lact-lac-B-T will increase the 10h-fasting Lac, such an increase will lead to negative concentrations of Lactate in the brain (N) or other tissues (T). We make a minor adjustment to Lac by setting Vdif-lact-Lac-B-L to 12*0.08 (the default value is 12). Details about the equations and parameters related to Pyr and Lac can be found in [1].**

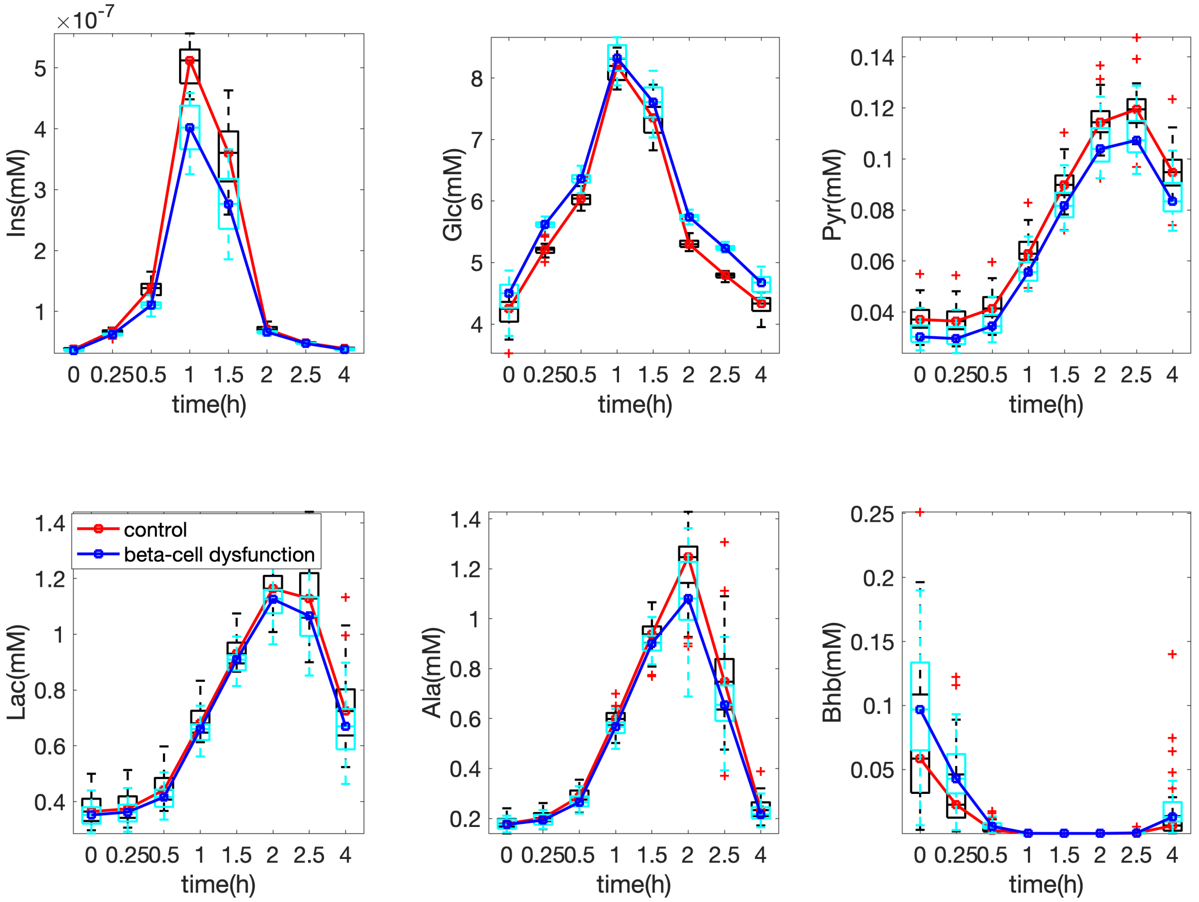

Figure S2.3: **Median time profiles of 50 control vs. 50 *beta-cell dysfunction* subjects for the *full-dynamic* data with individual variations introduced by setting the random perturbation level to $\alpha = 0.2$.**

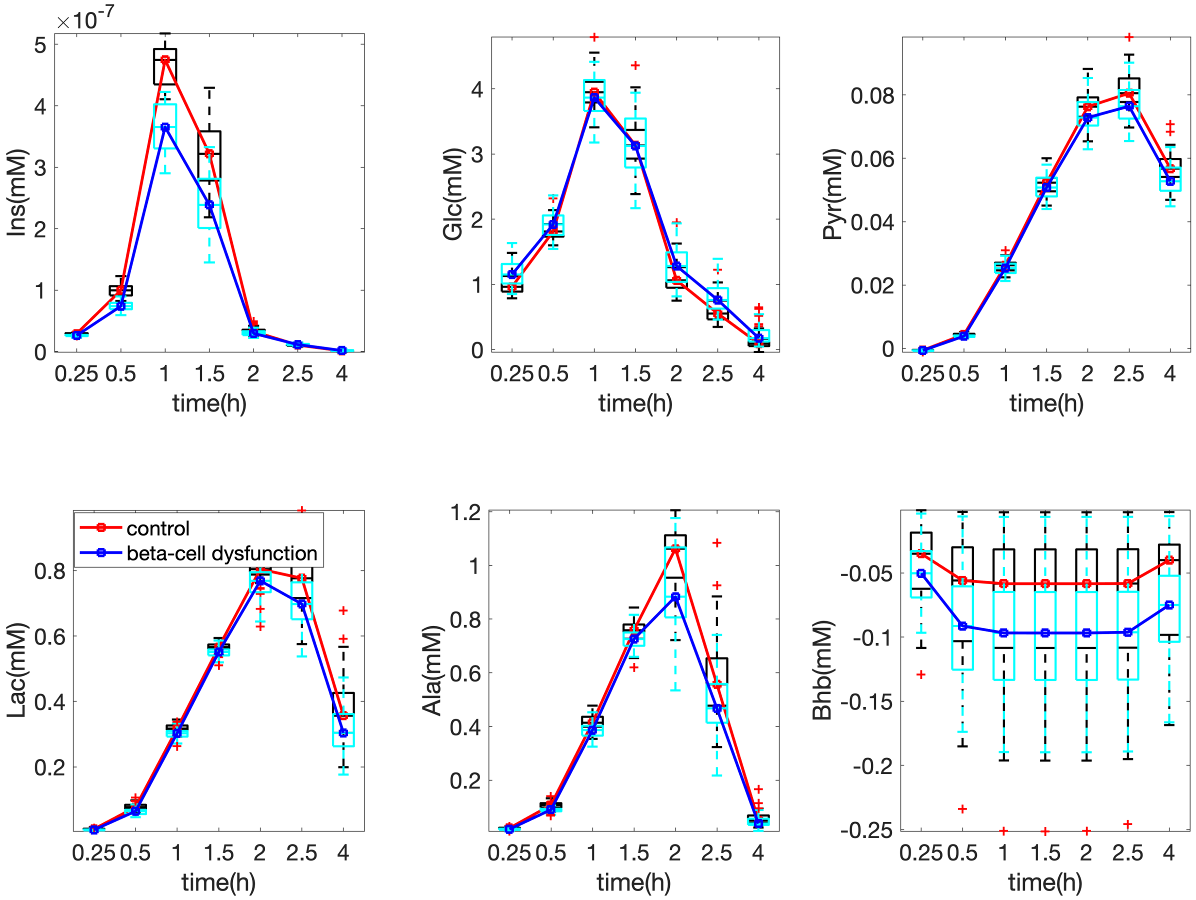

Figure S2.4: **Median time profiles of 50 control vs. 50 *beta-cell dysfunction* subjects for the *T0-corrected* data with individual variations introduced by setting the random perturbation level to $\alpha = 0.2$.**

Supplementary Tables

	glucose (g)	fat (g)	protein (g)
real data	60	75	20
simulated data	87	33	0

Table S2.1: **Meal compositions of the real and simulated data.**

	**Individual Variation**		**R**	**FMS**
insulin resistance	$\alpha = 0.2$	balanced	4	-
		unbalanced	4	1.00
	$\alpha = 0.4$	balanced	4	0.97
		unbalanced	4	0.97
beta-cell dysfunction	$\alpha = 0.2$	balanced	4	-
		unbalanced	4	1.00
	$\alpha = 0.4$	balanced	5	0.93
		unbalanced	5	0.90

Table S2.2: ***Full-dynamic data analysis.* Similarity between the CP factors (in the *metabolites* and *time* modes) extracted from different data sets and the CP factors of the data set with $\alpha = 0.2$ and balanced samples. Here, α denotes the level of individual variation, where a smaller number indicates a lower level of individual variation.**

	**Individual Variation**		**R**	**FMS**
insulin resistance	$\alpha = 0.2$	balanced	4	-
		unbalanced	4	1.00
	$\alpha = 0.4$	balanced	4	0.99
		unbalanced	4	0.99
beta-cell dysfunction	$\alpha = 0.2$	balanced	4	-
		unbalanced	4	0.96
	$\alpha = 0.4$	balanced	5	0.95
		unbalanced	5	0.95

Table S2.3: ***T0-corrected data analysis.* Similarity between the CP factors (in the *metabolites* and *time* modes) extracted from different data sets and the CP factors of the data set with $\alpha = 0.2$ and balanced samples. Here, α denotes the level of individual variation, where a smaller number indicates a lower level of individual variation.**

References

[1] Hiroyuki Kurata. Virtual metabolic human dynamic model for pathological analysis and therapy design for diabetes. *iScience*, 24(2):102101, 2021.
