# Supplementary material for: Analyzing postprandial metabolomics data using multiway models: a simulation study
Source: BMC Bioinformatics. 2024 Mar 4;25:94. doi: 10.1186/s12859-024-05686-w (PMC10913623; doi:10.1186/s12859-024-05686-w)
Supplement: Supplementary file 3 — Additional file 3. Selection of the number of components in CP models. [file 12859_2024_5686_MOESM3_ESM.pdf]

Selection of the number of components in CP models

When selecting the number of components (i.e., R) in CP models, we check the increase in model fit, drop in core consistency, and replicability of the model, which are discussed in the following. In addition, the selection also depends on the interpretability of the model.

Model fit

The *model fit* is defined as follows:

$$\text{Fit (\%)} = (1 - \frac{\left\| \mathscr{X} - \hat{\mathscr{X}} \right\|^2}{\| \mathscr{X} \|^2}) \times 100,$$

where $\mathscr{X}$ and $\hat{\mathscr{X}}$ correspond to the data tensor and approximation of the data by the model, respectively. A fit value close to 100% means that data $\mathscr{X}$ is well explained by the model; otherwise, there is an unexplained part left in the residuals. If a significant gain in model fit is observed as the number of components increases, we should consider having more components.

Core consistency

The core consistency diagnostic [1] is another approach for determining the number of components in a CP model. The core consistency compares the core array of the CP model with the core array obtained by modeling the data using a Tucker3 model [2] based on the CP factors. A core consistency value close to 100% indicates an appropriate number of components, while too many components may lead to a drop in the core consistency value.

Replicability

Another approach to selecting the number of components in a CP model is to check whether the model produces identical patterns from independent subsamples of the data set, e.g., as in split-half analysis [3]. Here, we propose a method to check the replicability of a model, which can be viewed as an extension of split-half analysis. The replicability is tested by leaving out random subsets of subjects (randomly selecting and leaving out 10% of the total subjects) and computing the similarity of the factors in the *metabolites* and *time* modes extracted using a CP model from the remaining data. We quantify the similarity of CP models from two splits using the factor match score (FMS) introduced in the main text. The model is considered replicable if the patterns are similar enough. Figure S3.1 illustrates the proposed replicability check.

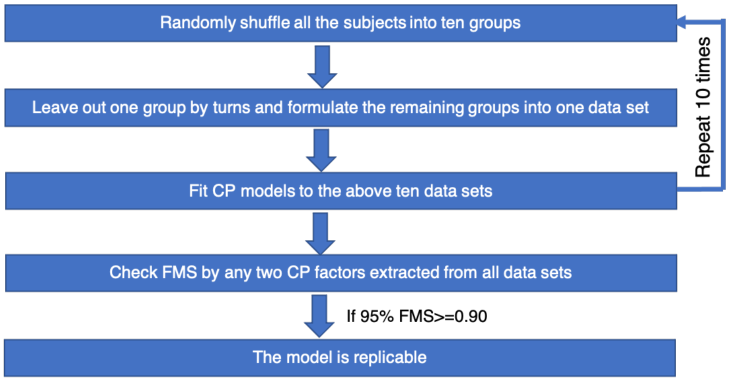

Figure S3.1: **The replicability test used to select the number of components.**

Model selection for each data set

Full-dynamic analysis for the data with *insulin-resistant* vs. control group, $\alpha = 0.2$, balanced samples

The model fit increases evidently from $R = 1$ to $R = 3$ (see Figure S3.2a). The core consistency drops significantly when we increase the number of components from $R = 5$ to $R = 6$ (see Figure S3.2b).In addition, the 6-component model can not be replicated (see Figure S3.3f). Therefore, we select among models with $R \leq 5$. We choose the 4-component model since it is more interpretable and can be replicated (see Figure S3.3d). Compared with the 3-component model, the 4-component model captures an extra constant temporal pattern (i.e., the fourth component), which reflects the fasting-state information (see the comparison of Figure S3.4 and S3.5). The 5-component model splits one of the patterns in the 4-component model, i.e., the first component ($\mathbf{a}_1$, $\mathbf{b}_1$ and $\mathbf{c}_1$ in Figure S3.5), where Lac and Ala have large coefficients on $\mathbf{b}_1$, into two components, i.e., the first and fifth components in the 5-component model (Figure S3.6).

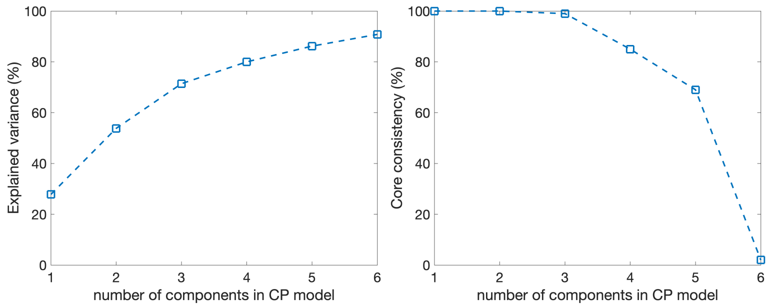

(a) (b)

Figure S3.2: **(a) Model fit, and (b) core consistency of CP models using different numbers of components for the *full-dynamic* data with *insulin-resistant* vs. control group, $\alpha = 0.2$ and balanced samples.**

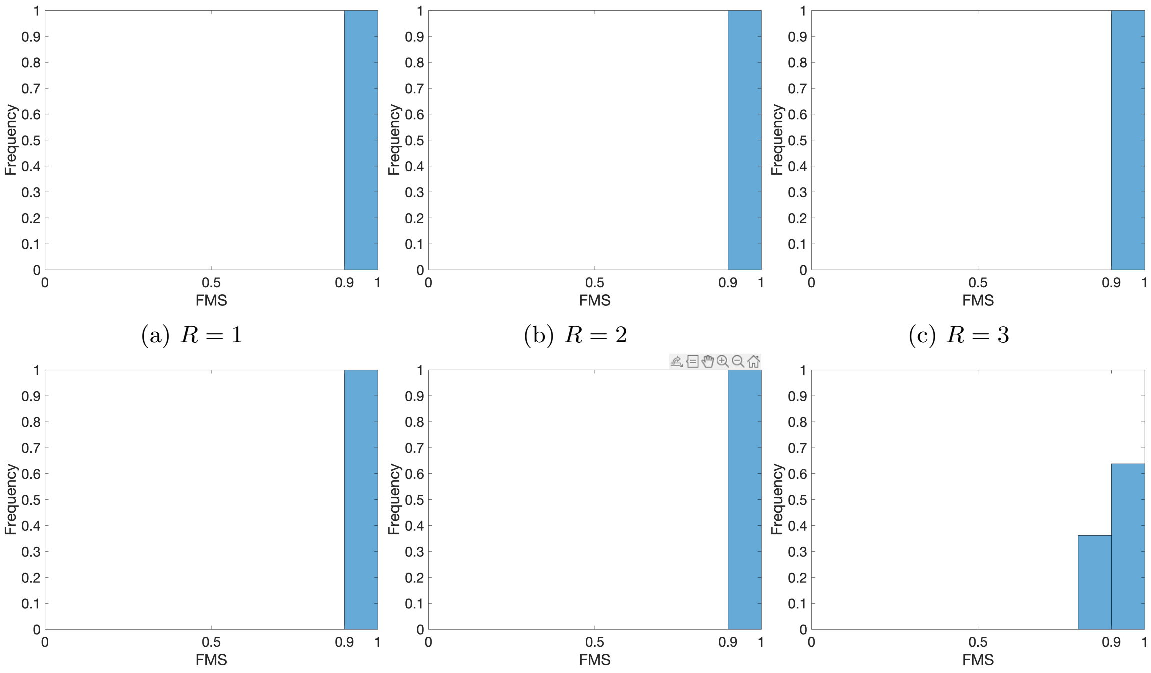

(a) $R = 1$ (b) $R = 2$ (c) $R = 3$

(d) $R = 4$ (e) $R = 5$ (f) $R = 6$

Figure S3.3: **Histogram of FMS values between CP factors (in the *metabolites* and *time* modes) extracted from all splits of the *full-dynamic* data with *insulin-resistant* vs. control group, $\alpha = 0.2$ and balanced samples.**

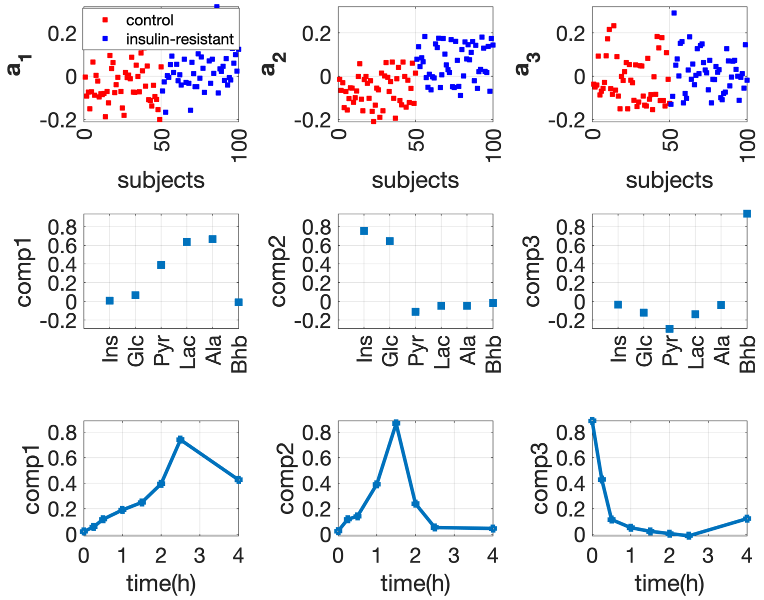

Figure S3.4: **Factors of the 3-component CP model for the *full-dynamic* data with *insulin-resistant* vs. control group and $\alpha = 0.2$.**

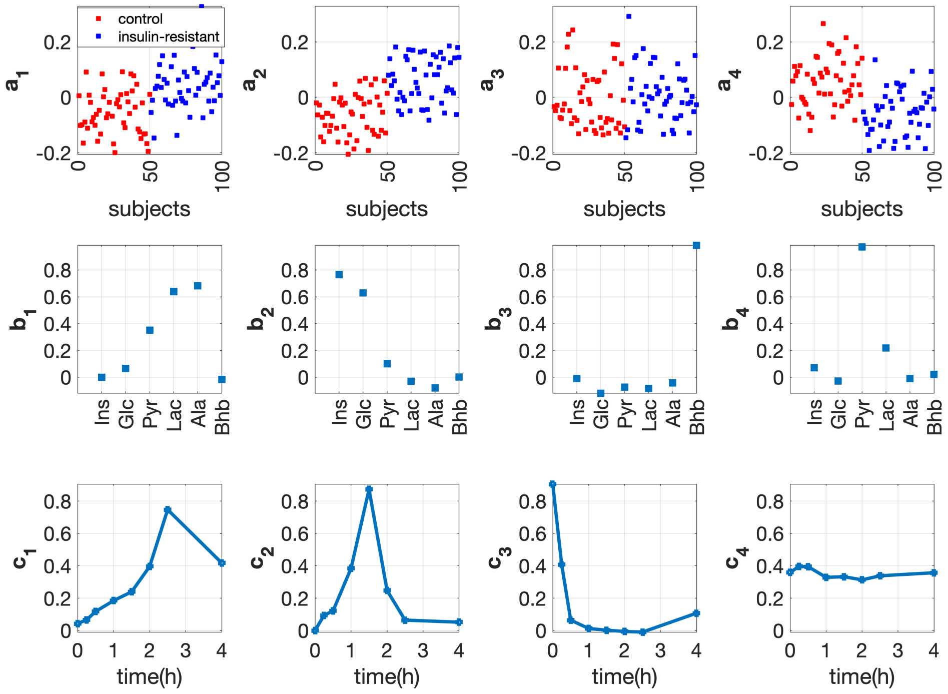

Figure S3.5: **Factors of the 4-component CP model for the *full-dynamic* data with *insulin-resistant* vs. control group and $\alpha = 0.2$.**

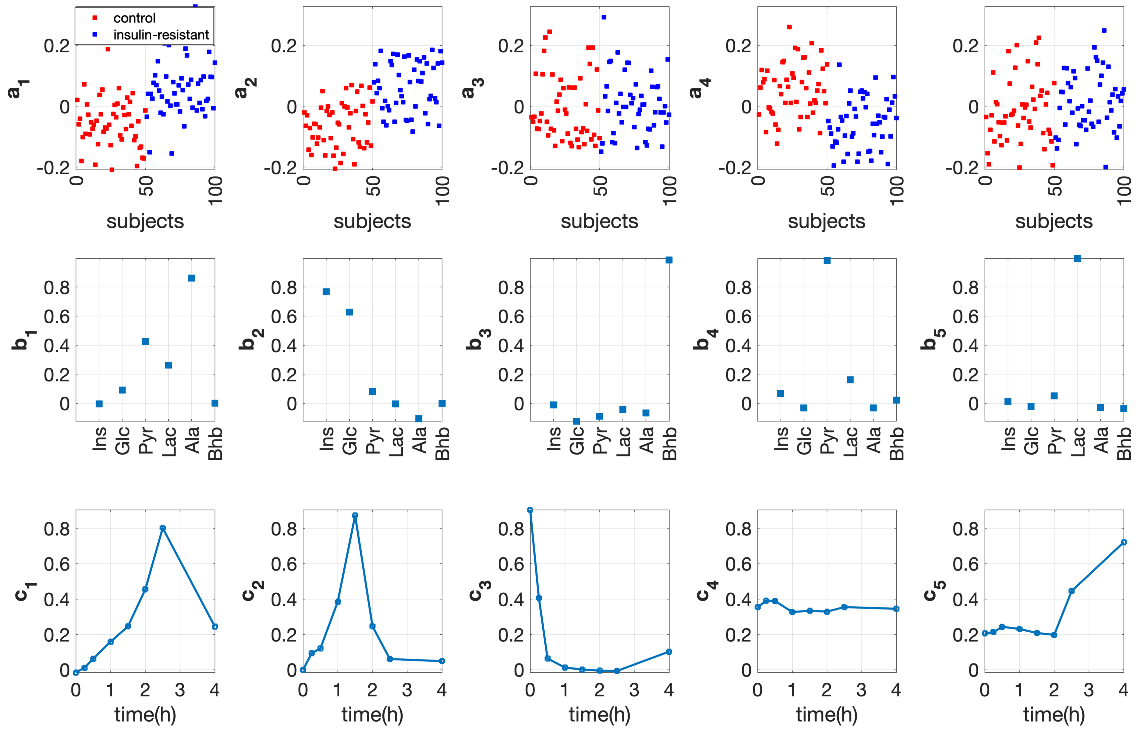

Figure S3.6: **Factors of the 5-component CP model for the *full-dynamic* data with *insulin-resistant* vs. control group and $\alpha = 0.2$.**

T0-corrected analysis for the data with *insulin-resistant* vs. control group, $\alpha = 0.2$ and balanced samples

The model fit increases evidently from $R = 1$ to $R = 3$ (see Figure S3.7a). The core consistency drops evidently when we increase the number of components from $R = 5$ to $R = 6$ (see Figure S3.7b); therefore, we consider models with $R \leq 5$. The 5-component model cannot be replicated but the 4-component model can (see Figure S3.8). Therefore, we may prefer the 4-component model. In addition, compared with the 3-component model, the 4-component model captures an extra dynamic pattern related to metabolite Pyr (see the comparison of Figure S3.9 with S3.10), which provides useful information in terms of understanding metabolic differences at the *fasting* vs. *pure-dynamic* state.

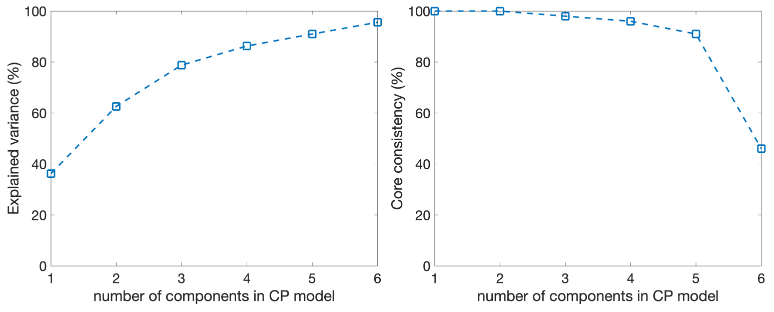

(a) (b)

Figure S3.7: **(a) Model fit, and (b) core consistency of the CP models using different numbers of components for *T0-corrected* data generated with *insulin-resistant* vs. control group, $\alpha = 0.2$ and balanced samples.**

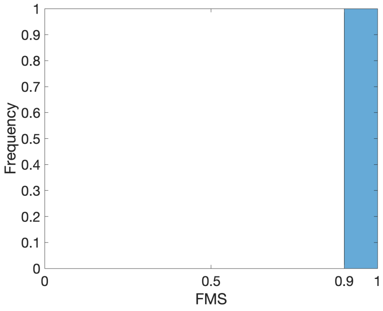

(a) $R = 1$

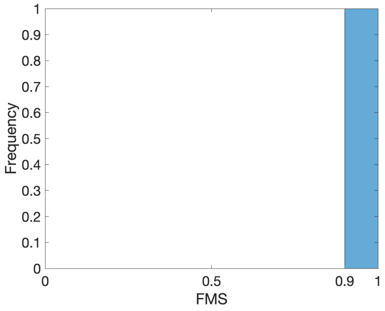

(b) $R = 2$

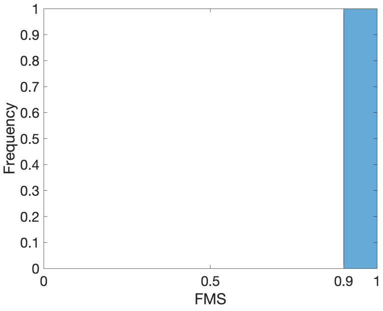

(c) $R = 3$

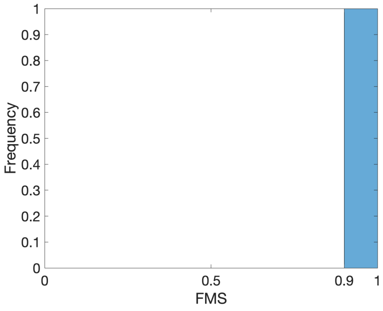

(d) $R = 4$

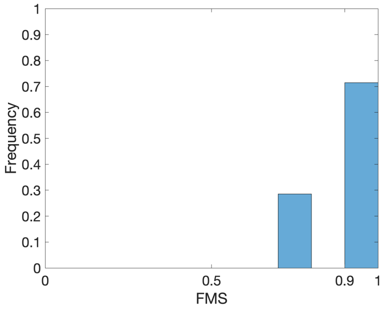

(e) $R = 5$

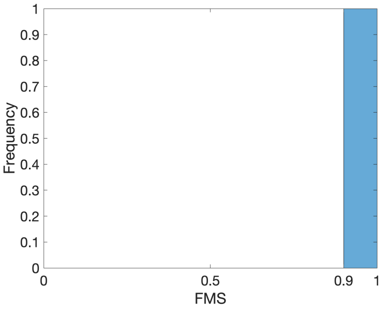

(f) $R = 6$

Figure S3.8: **Histogram of FMS values between CP factors (in the *metabolites* and *time* modes) extracted from all splits of *T0-corrected* data with *insulin-resistant* vs. control group, $\alpha = 0.2$ and balanced samples.**

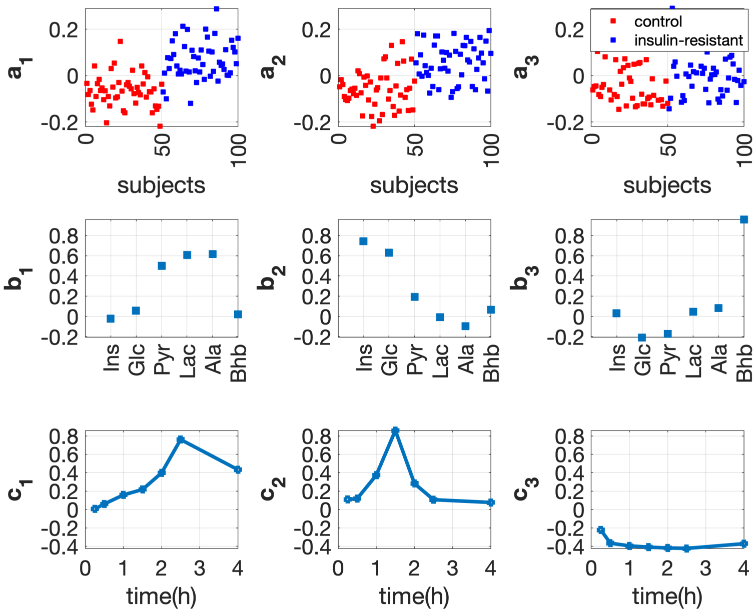

Figure S3.9: **Factors of the 3-component CP model for the *T0-corrected* data with *insulin-resistant* vs. control group and $\alpha = 0.2$.**

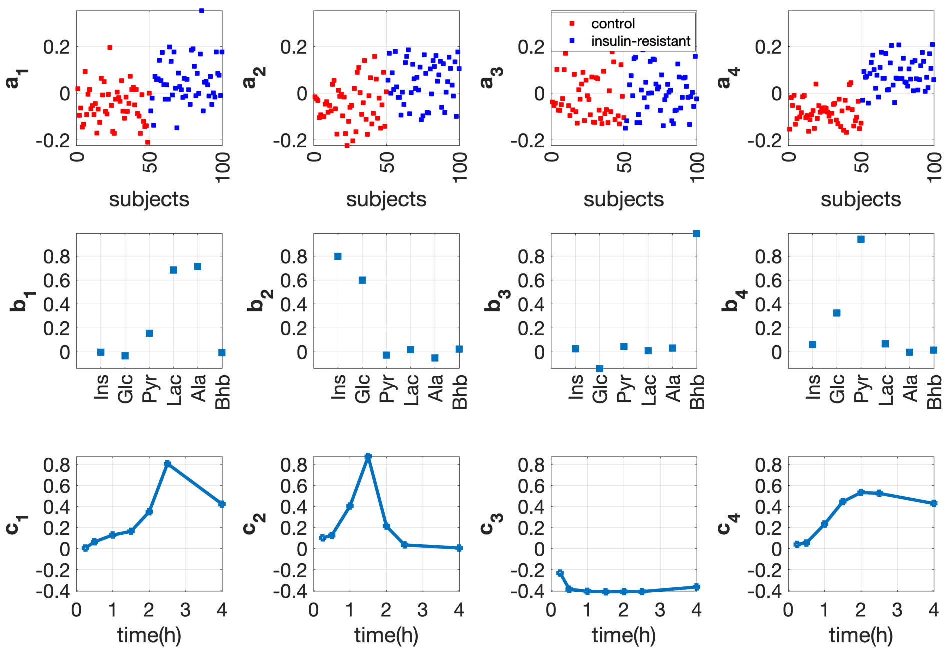

Figure S3.10: **Factors of the 4-component CP model for the *T0-corrected* data with *insulin-resistant* vs. control group and $\alpha = 0.2$.**

Full-dynamic* analysis for the data generated with *beta-cell dysfunction* vs. control group, $\alpha = 0.2$ and balanced samples

The model fit increases evidently from $R = 1$ to $R = 4$ (see Figure S3.11a). The core consistency drops clearly when we increase the number of components from $R = 4$ to $R = 5$ and from $R = 5$ to $R = 6$ (see Figure S3.11b). This indicates considering CP models with $R = 4$ or $R = 5$. The 5-component model cannot be replicated but the 4-component model can (see Figure S3.12). Therefore, we select the 4-component model. In addition, compared with the 3-component model, the 4-component model captures the subject group separation much better (see Figure S3.13 vs. S3.14).

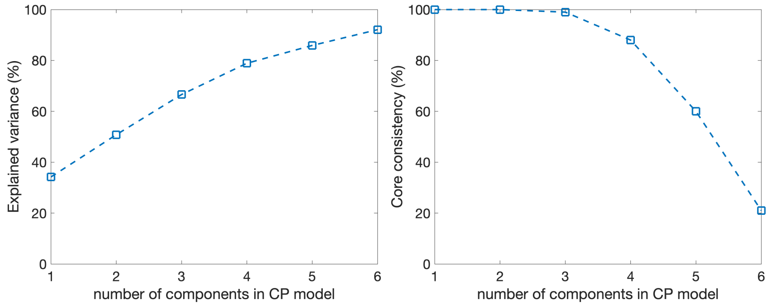

(a) (b)

Figure S3.11: **(a) Model fit, and (b) core consistency of the CP models using different numbers of components for the (*full-dynamic*) data with *beta-cell dysfunction* vs. control group, $\alpha = 0.2$ and balanced samples.**

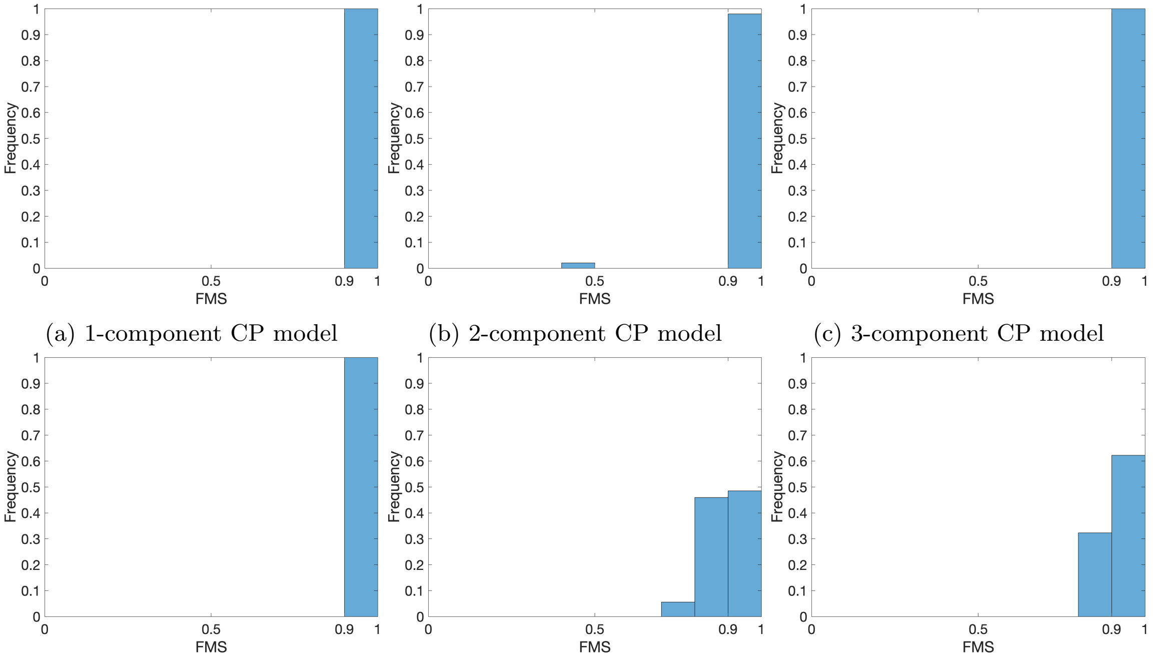

(a) 1-component CP model (b) 2-component CP model (c) 3-component CP model

(d) $R = 4$ (e) $R = 5$ (f) $R = 6$

Figure S3.12: **Histogram of FMS values between CP factors (in the *metabolites* and *time* modes) extracted from all splits of *full-dynamic* data with *beta-cell dysfunction* vs. control group, $\alpha = 0.2$ and balanced samples.**

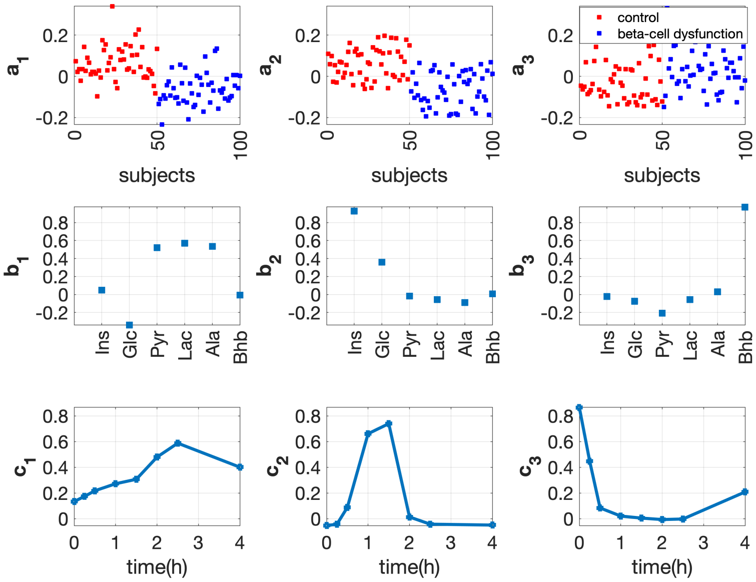

Figure S3.13: **Factors of the 3-component CP model for the *full-dynamic* data with *beta-cell dysfunction* vs. control group, $\alpha = 0.2$ and balanced samples.**

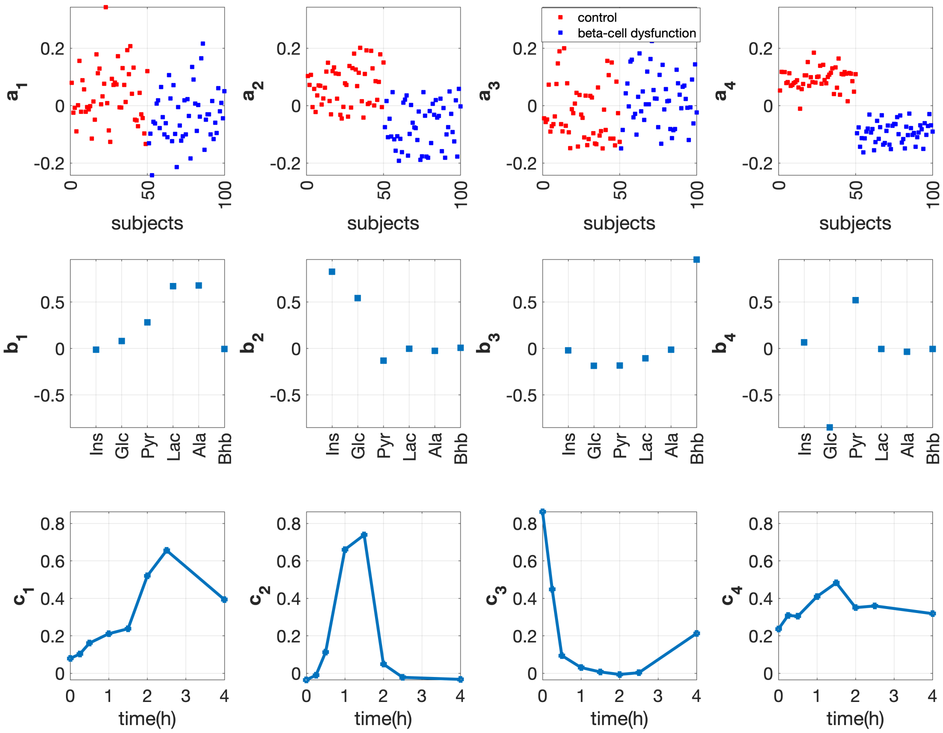

Figure S3.14: **Factors of the 4-component CP model for the *full-dynamic* data with *beta-cell dysfunction* vs. control group, $\alpha = 0.2$ and balanced samples.**

T0-corrected analysis for the data with *beta-cell dysfunction* vs. control group, $\alpha = 0.2$ and balanced samples

The model fit increases evidently from $R = 1$ to $R = 3$ (see Figure S3.15a). The core consistency drops clearly when we increase the number of components from $R = 3$ to $R = 4$ and from $R = 5$ to $R = 6$ (see Figure S3.15b). Therefore, we consider models with $R = 3$, $R = 4$, or $R = 5$. The replicability check shows that both the 3-component and 5-component models can be reproduced well (see Figure S3.16c and S3.16e). For the 4-component model, a few splits have different patterns from the remaining splits, as illustrated in Figure S3.16d. However, the 4-component model is more interpretable than the 3-component and 5-component models. Compared with the 3-component model, the 4-component model extracts Bhb out from the second component ($\mathbf{b}_2$) in the 3-component model in Figure S3.17, which indicates that Bhb does not contribute to the subject group separation. The 5-component model splits the fourth component ($\mathbf{a}_4$, $\mathbf{b}_4$ and $\mathbf{c}_4$) in the 4-component model into two components (the fourth and fifth components in the 5-component model), which does not reveal that Glc and Pyr are close to each other (see Figure S3.18 vs. S3.19). Therefore, we prefer the 4-component model for this data set.

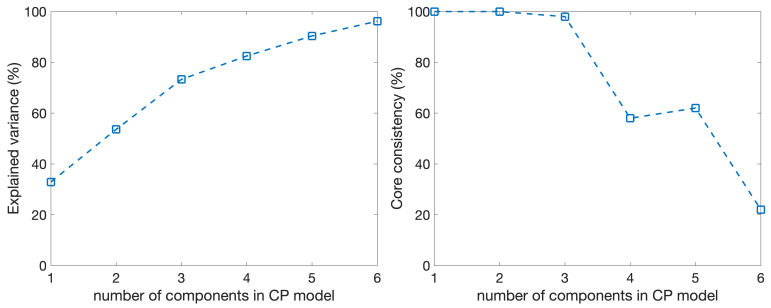

(a) (b)

Figure S3.15: **(a) Model fit, and (b) core consistency of the CP models using different numbers of components for the *T0-corrected* data with *beta-cell dysfunction* vs. control group, $\alpha = 0.2$ and balanced samples**

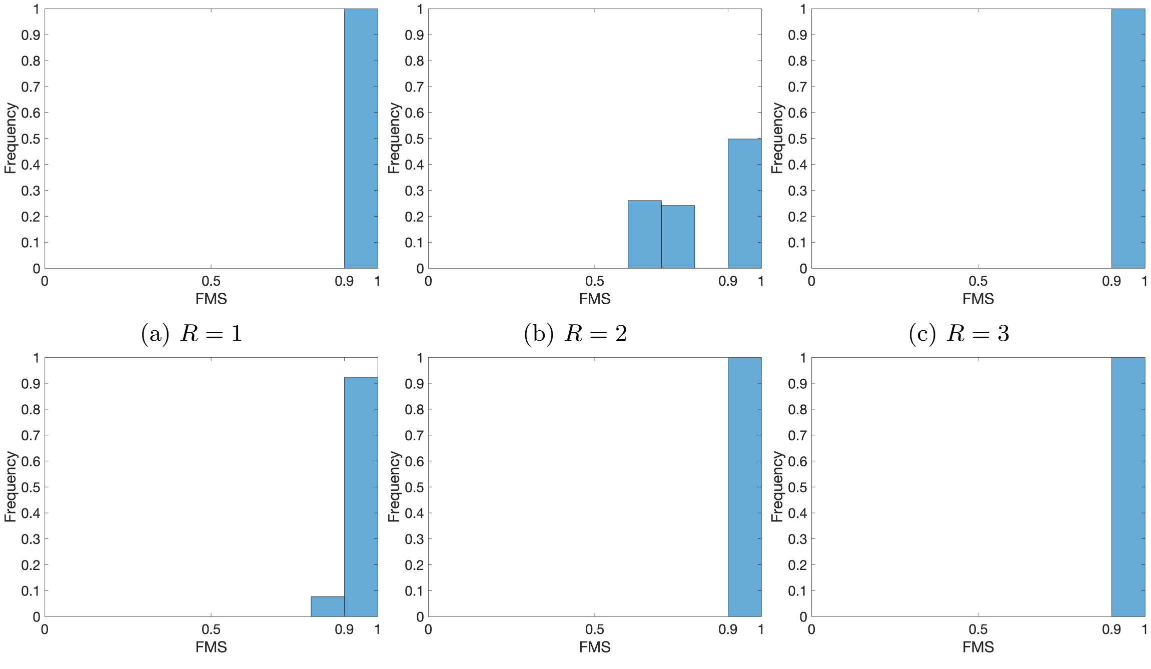

(a) $R = 1$ (b) $R = 2$ (c) $R = 3$

(d) $R = 4$ (e) $R = 5$ (f) $R = 6$

Figure S3.16: **Histogram of FMS values between CP factors (in the *metabolites* and *time* modes) extracted from all splits of the *T0-corrected* data with *beta-cell dysfunction* vs. control group, $\alpha = 0.2$ and balanced samples.**

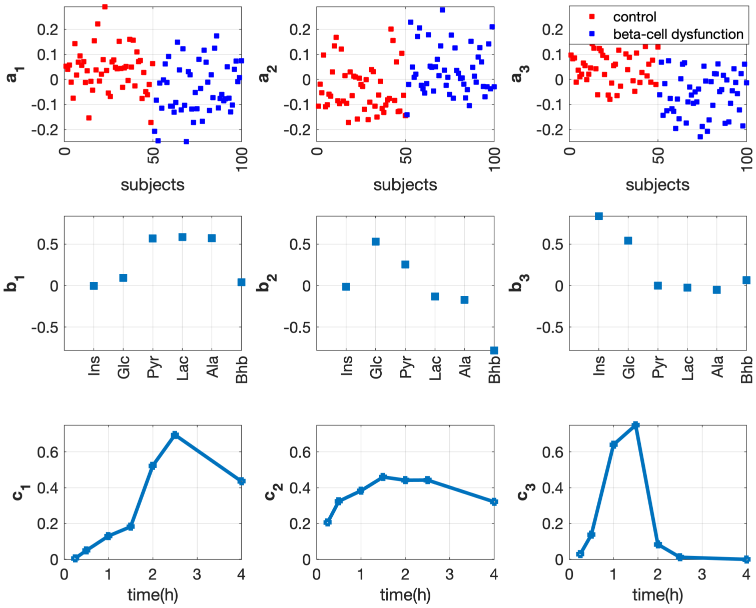

Figure S3.17: **Factors of the 3-component CP model for the *T0-corrected* data with *beta-cell dysfunction* vs. control group and $\alpha = 0.2$.**

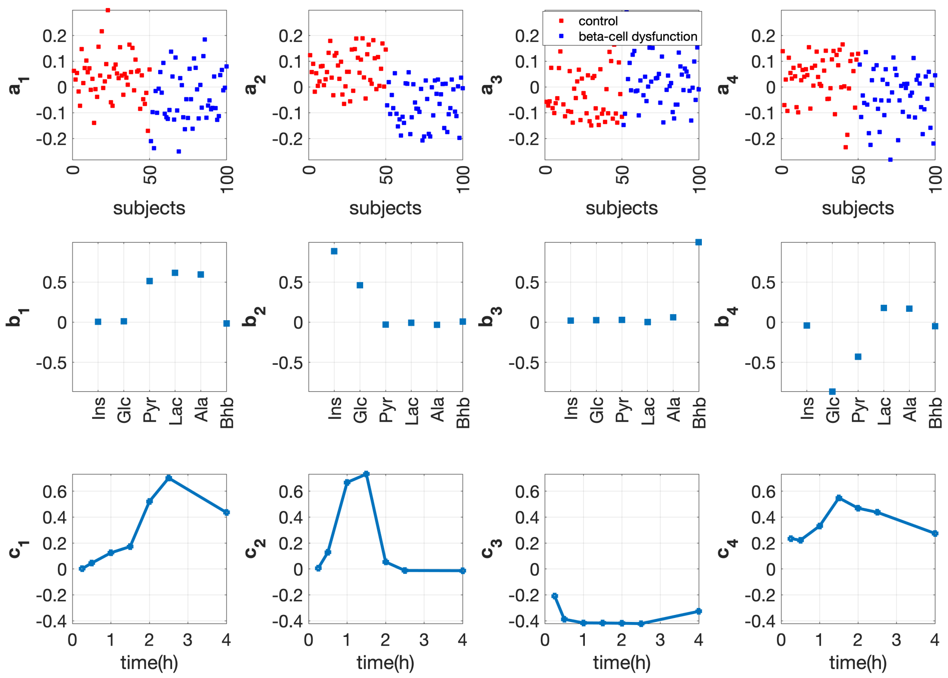

Figure S3.18: **Factors of the 4-component CP model for the *T0-corrected* data with *beta-cell dysfunction* vs. control group and $\alpha = 0.2$.**

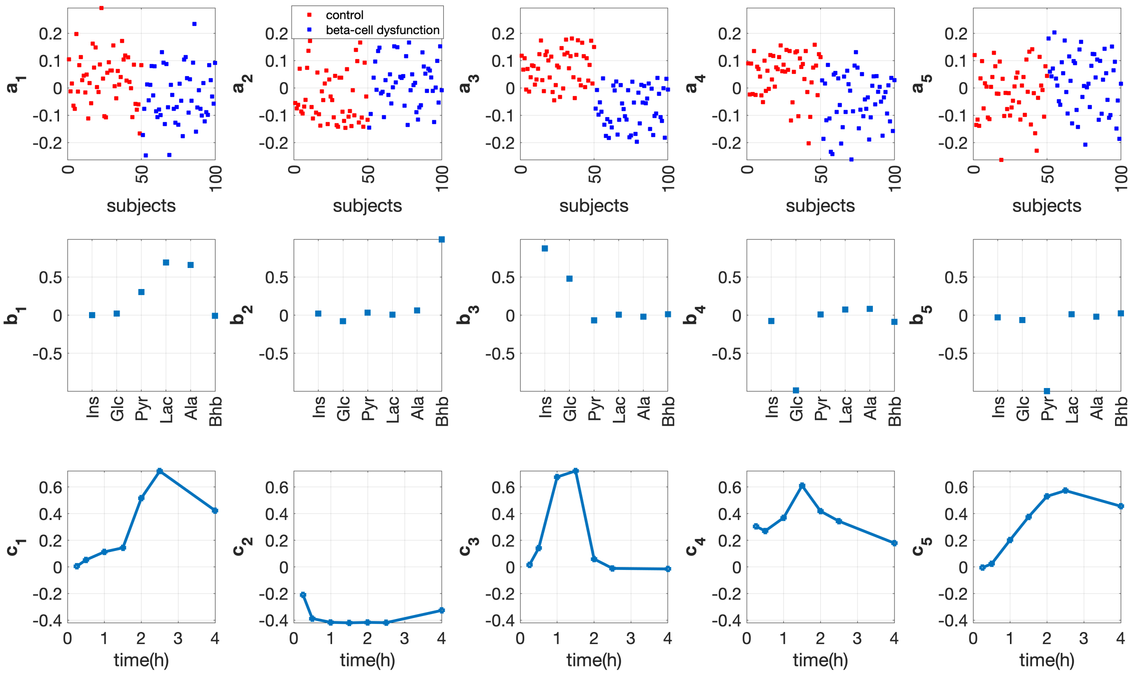

Figure S3.19: **Factors of the 5-component CP model for the *T0-corrected* data with *beta-cell dysfunction* vs. control group and $\alpha = 0.2$.**

Full-dynamic **analysis for the real data consisting of 299 subjects and 6 measurements**

The model fit increases evidently from $R = 1$ to $R = 3$ (Figure S3.20a). The core consistency drops significantly when we increase the number of components from $R = 3$ to $R = 4$ and from $R = 4$ to $R = 5$ (Figure S3.20b). This indicates considering the models with $R = 3$ or $R = 4$. The 3-component model can be well replicated, and there are some unlucky splits when using the 4-component model (see Figure S3.21). The main differences between the 4-component model from the 3-component model are Ala splits

out from the third component in the 3-component model, and Pyr splits from the first component in the 3-component model to the third component in the 4-component model (see Figure S3.22 vs. S3.23). However, it makes sense that Pyr, Lac and Ala stay close since they are tied to each other with reactions Pyr↔Lac and Pyr↔Ala as shown in the pathway in the main text. Therefore, we prefer the 3-component CP model for this analysis.

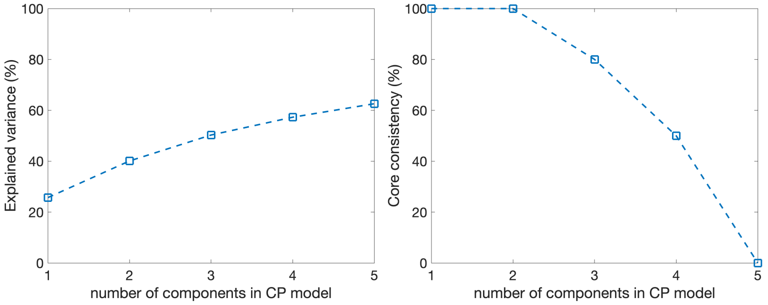

(a) (b)

Figure S3.20: **(a) Model fit, and (b) core consistency of the CP models using different numbers of components for the real (*full-dynamic*) data set with 299 subjects and 6 metabolites.**

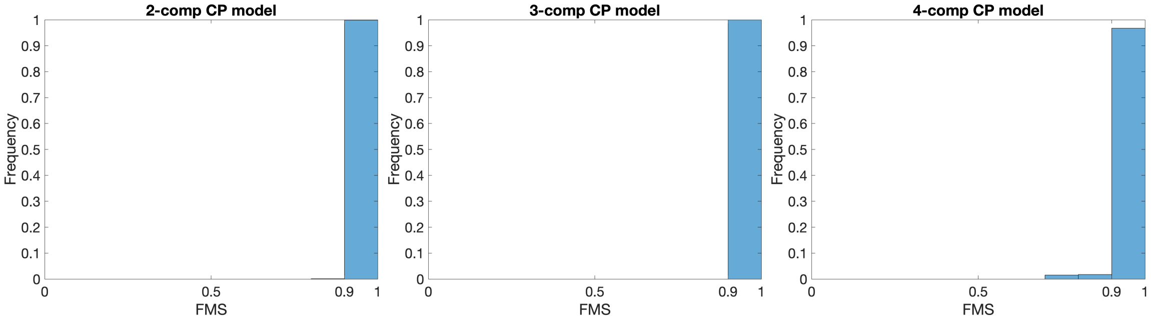

(a) $R = 2$ (b) $R = 3$ (c) $R = 4$

Figure S3.21: **Histogram of FMS values between CP factors (in the *metabolites* and *time* modes) extracted from all splits of the real *full-dynamic* data consisting of 6 measurements.**

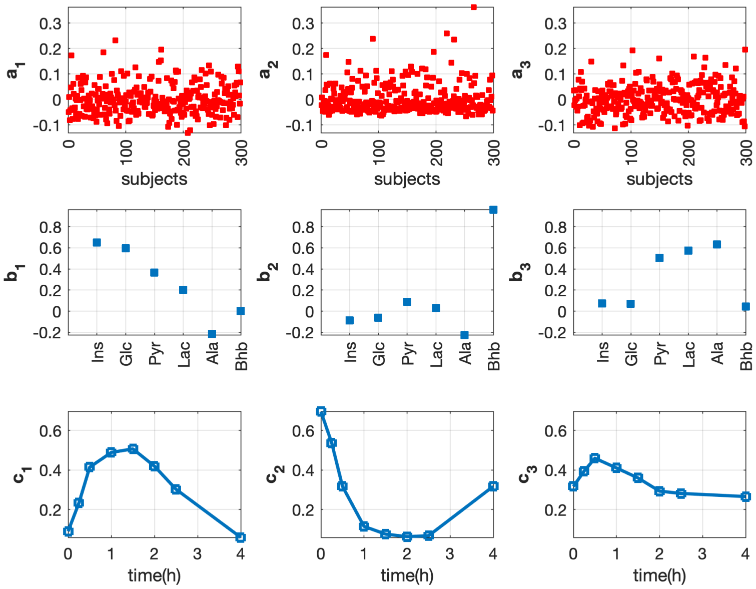

Figure S3.22: **Factors of the 3-component CP model for the real data set with 6 measurements.**

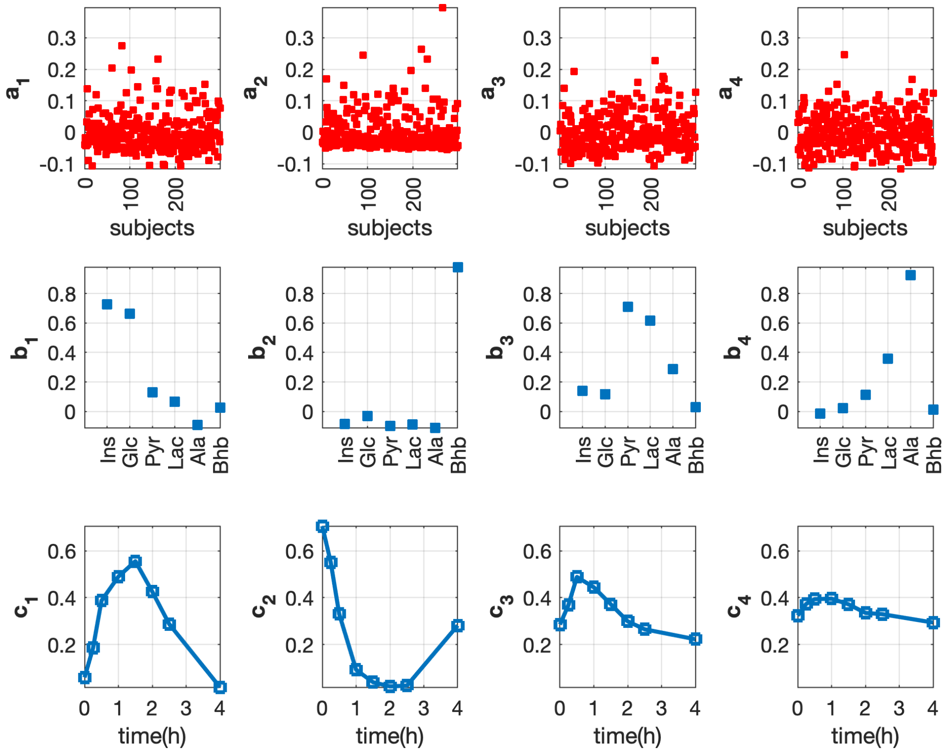

Figure S3.23: **Factors of the 4-component CP model for the real data set with 6 measurements.**

References

[1] Rasmus Bro and Henk AL Kiers. A new efficient method for determining the number of components in parafac models. *Journal of Chemometrics*, 17(5):274–286, 2003.

[2] Ledyard R Tucker. Some mathematical notes on three-mode factor analysis. *Psychometrika*, 31(3):279–311, 1966.

[3] R. A. Harshman and W. S. De Sarbo. An application of PARAFAC to a small sample problem, demonstrating preprocessing, orthogonality constraints, and split-half diagnostic techniques in *Research Methods for Multimode Data Analysis. Praeger: New York*, pages 602–642, 1984.
